# Improving the public health utility of global cardiovascular mortality data: the rise of ischemic heart disease

**DOI:** 10.1186/1478-7954-9-8

**Published:** 2011-03-15

**Authors:** Ryan M Ahern, Rafael Lozano, Mohsen Naghavi, Kyle Foreman, Emmanuela Gakidou, Christopher JL Murray

**Affiliations:** 1 Institute for Health Metrics and Evaluation, 2301 Fifth Ave., Suite 600, Seattle, WA 98121, USA

## Abstract

**Background:**

High-quality, cause-specific mortality data are critical for effective health policy. Yet vague cause of death codes, such as heart failure, are highly prevalent in global mortality data. We propose an empirical method correcting mortality data for the use of heart failure as an underlying cause of death.

**Methods:**

We performed a regression analysis stratified by sex, age, and country development status on all available ICD-10 mortality data, consisting of 142 million deaths across 838 country-years. The analysis yielded predicted fractions with which to redistribute heart failure-attributed deaths to the appropriate underlying causes of death. Age-adjusted death rates and rank causes of death before and after correction were calculated.

**Results:**

Heart failure accounts for 3.1% of all deaths in the dataset. Ischemic heart disease has the highest redistribution proportion for ages 15-49 and 50+ in both sexes and country development levels, causing gains in age-adjusted death rates in both developed and developing countries. COPD and hypertensive heart disease also make significant rank gains. Reproductive-aged women in developing country-years yield the most diverse range of heart failure causes.

**Conclusions:**

Ischemic heart disease becomes the No. 1 cause of death in several developed countries, including France and Japan, underscoring the cardiovascular epidemic in high-income countries. Age-adjusted death rate increases for ischemic heart disease in low- and middle-income countries, such as Argentina and South Africa, highlight the rise of the cardiovascular epidemic in regions where public health efforts have historically focused on infectious diseases. This method maximizes the use of available data, providing better evidence on major causes of death to inform policymakers in allocating finite resources.

## Introduction

Accurate cause of death data are critical to developing an effective health policy agenda. Just as clinicians must accurately diagnose patients before prescribing treatment, policymakers must assess population-level disease burden in order to prioritize health interventions [1]. Despite this, the availability of high-quality, cause-specific mortality data is limited [2-6]. Even for countries with high vital registration coverage, the quality of the data is often substandard [2,4-7]. Using the heart failure cause of death code as a case study, this article proposes a method to improve the quality of vital registration mortality data to provide policymakers with more accurate diagnostic datasets from which to prioritize health interventions.

In mortality data analysis, the underlying cause of death is of greatest importance [8,9]. The World Health Organization (WHO) defines the underlying cause of death as "the disease or injury which initiated the train of morbid events leading directly to death, or the circumstance of the accident or violence which produced the fatal injuries" [8]. The underlying cause must satisfy two criteria fundamental to this definition. Specifically, the underlying cause must be the primary or initial cause such that it represents a logical stage of intervention to prevent death, while retaining its status as a disease or injury to which death can be definitively assigned. One of the most significant barriers to accurate cause of death data is the widespread use of nonspecific cause of death codes, such as those for heart failure. These ambiguous or vague codes were first described as "garbage codes" in the Global Burden of Disease (GBD) 1990 study, in reference to ill-defined cardiovascular, malignancy, and injury codes [2]. A recent paper by Naghavi et al stresses that garbage codes negatively impact the public health utility of cause of death data [10]. This problem stems from the fact that the International Classification of Diseases (ICD), the coding scheme used in most countries with vital registration systems, contains many codes that signify signs, symptoms, and conditions, or intermediate and immediate causes of death [9]. These codes are often misrecorded as an underlying cause of death on death certificates or in mortality datasets for a number of reasons [11-15]. Physicians do not always have access to accurate or complete medical records from which to determine the correct underlying cause of death [6,15,16]. They may be inadequately trained to complete death certificates [17], or, in some cases, may intentionally miscode deaths in the face of political, financial, or social pressure (i.e., HIV-related stigma). In developing nations, these realities are compounded by the fact that only a small percentage of death certificates are completed by physicians [15].

Heart failure is the end stage of many cardiac and noncardiac pathological processes, from ischemic heart disease and the range of cardiomyopathies to respiratory disease and severe anemia. As such, heart failure is not an underlying cause of death according to the WHO definition, but rather an intermediate cause of death with a diverse range of possible underlying causes of death. The use of heart failure as an underlying cause of death code leads to a vague and sometimes inaccurate representation of the population-level causes of death [2,18]. Because prevention, detection, and treatment efforts for severe anemia and ischemic heart disease are different, it is essential for policymakers to know the true etiology of heart failure-attributed deaths. [Please note that for the rest of the paper, the term "heart failure-attributed deaths" refers solely to deaths in which heart failure was attributed as the underlying cause of death and not deaths in which heart failure is coded as a complication of the underlying cause in Part I of the death certificate or contributory causes in Part II of the death certificate.] In fact, Naghavi et al defines garbage codes, of which heart failure is most frequent, as "all deaths assigned to codes that should be redistributed to enhance the validity of public health analysis" [10]. A process called heart failure redistribution, in which heart failure-attributed deaths in existing mortality data are reallocated to the correct underlying causes of death, can accomplish this.

Though the problem of miscoded mortality data is appreciated in the literature [2-6,10,12,14-20], there is a paucity of redistribution methods described in the literature. Two methods used in the GBD literature are outlined below. GBD 1990 and 2001 redistributed aggregated groups of ill-defined cardiovascular codes (heart failure, atherosclerosis, essential hypertension, etc.) to one underlying cause of death, ischemic heart disease [2,4,18]. Less specific garbage codes, such as "other ill-defined, and unspecified causes of mortality (ICD-10, R99)," have been redistributed proportionally across all-cause mortality [2].

Building on these previous approaches, this paper describes in detail a novel method to carry out heart failure redistribution. This method was employed, but not described, in Naghavi's recent work [10]. The method redistributes from a single garbage code - heart failure - to multiple underlying causes of death while accounting for the fact that clinicians miscode heart failure deaths at rates *dis*proportionate to the relative prevalence of its plausible underlying causes. It is important to note here that this method does *not *redistribute heart failure-attributed deaths proportionally across the underlying causes, a method that has been used in the past. We then compare cause of death patterns on a pre- and post-redistribution mortality dataset to show the effect of heart failure redistribution on age-adjusted death rates and cause of death rankings for the leading causes of death in 2005.

## Methods

### Data

The mortality data used in our model are a combination of the Sept. 8, 2009 version of the WHO mortality database [21] and additional country-years obtained by the Institute for Health Metrics and Evaluation. No primary data were collected for this study and all data were completely anonymous. Using the ICD coding scheme and listed by country-year, the WHO mortality database is a publicly available dataset listing the number of underlying deaths due to each ICD code stratified by age and sex. The additional country-year data used in the model are formatted in a similar manner. Country-years used in the model were restricted to ICD-10, the most recent iteration of the ICD. The dataset used in the model consists of 141,940,984 deaths across 838 country-years ranging from 1994-2007, representing 115 countries and 17 of the 21 GBD 2005 regions. [Additional file 1 lists each of the countries in the dataset by GBD region, specifying the number and range of country-years.]

### Heart failure in the International Classification of Diseases

Heart failure is defined as the following ICD-10 codes: I50, I50.0, I50.1, I50.9:

• I50 Heart failure

• I50.0 Congestive heart failure

• I50.1 Left ventricular failure

• I50.9 Heart failure, unspecified

Because the ICD-10 is structured in increasing levels of specificity, I50 is a three-character code that encompasses each of the corresponding four-character codes. Most country-years in the dataset list deaths at the four-character level. Some country-years employ a lesser degree of specificity and list deaths at the three-character level. In this analysis, we have aggregated I50.0, I50.1, and I50.9 into one code and treated it as I50. Attempts to analyze codes at the four-character level yielded no significant differences. [Additional file 1 includes a column that describes, by country, the percentage of all heart failure-attributed deaths assigned to I50.9 for countries that list deaths at the four-character level.]

### Defining the target list

A list of potential underlying causes of death to which heart failure may be redistributed, known as targets, was chosen. This *a priori *conceptual decision was rooted in the pathophysiology of heart failure. We identified 49 causes at the three-character, ICD version 10 level. One four-character code was included in target group 10, I31.1 chronic constrictive pericarditis. The 49 causes are aggregated into 12 target groups on the basis of heart failure pathophysiology. The target groups are as follows:

• TG1 - Aortic aneurysm

• TG2 - Chronic obstructive pulmonary disease (COPD)

• TG3 - Cardiomyopathy

• TG4 - Chronic severe anemia

• TG5 - Congenital heart anomalies

• TG6 - Hypertensive heart disease

• TG7 - Ischemic heart disease

• TG8 - Other respiratory diseases

• TG9 - Other valve diseases

• TG10 - Pericarditis, endocarditis, myocarditis

• TG11 - Rheumatic heart disease

• TG12 - Thyroid disorders

Table 1 is a list of target groups used in this model and their corresponding ICD-10 codes. This target list is globally representative and does not include key regional causes, such as Chagas disease in Latin America, an important factor to consider in redistributing regional mortality datasets.

**Table 1:** Heart failure target list, by group

Target Group 1	Aortic Aneurysm
I71	Aortic aneurysm and dissection

**Target Group 2**	**COPD**

J43	Emphysema

J44	Other chronic obstructive pulmonary disease

**Target Group 3**	**Cardiomyopathy**

I42	Cardiomyopathy

**Target Group 4**	**Chronic Severe Anemias**

D50	Iron deficiency anemia

D55	Anemia due to enzyme disorders

D56	Thalassemia

D57	Sickle-cell disorders

D58	Other hereditary hemolytic anemias

D59	Acquired hemolytic anemia

**Target Group 5**	**Congenital Heart Anomalies**

Q20	Congenital malformations of cardiac chambers and connections

Q21	Congenital malformations of cardiac septa

Q22	Congenital malformations of pulmonary and tricuspid valves

Q23	Congenital malformations of aortic and mitral valves

Q24	Other congenital malformations of heart

Q25	Congenital malformations of great arteries

**Target Group 6**	**Hypertensive heart disease**

I11	Hypertensive heart disease

I12	Hypertensive renal disease

I13	Hypertensive heart and renal disease

**Target Group 7**	**Ischemic Heart Disease**

I21	Acute MI

I22	Subsequent MI

I23	Certain current complications following acute MI

I24	Other acute ischemic heart diseases

I25	Chronic ischemic heart disease

**Target Group 8**	**Other Respiratory Diseases**

J60	Coalworker's pneumoconiosis

J61	Pneumoconiosis due to asbestos or other mineral fibers

J62	Pneumoconiosis due to dust containing silica

J63	Pneumoconiosis due to other inorganic dusts

J64	Unspecified pneumoconiosis

J65	Pneumoconiosis associated with tuberculosis

**Target Group 9**	**Other valve diseases**

I34	Non-rheumatic mitral valve disorders

I35	Non-rheumatic aortic valve disorders

I36	Non-rheumatic tricuspid valve disorders

I37	Pulmonary valve disorders

**Target Group 10**	**Pericarditis, endocarditis, myocarditis**

I33	Acute and subacute endocarditis

I40	Acute myocarditis

I31.1	Chronic constrictive pericarditis

**Target Group 11**	**Rheumatic Heart Disease**

I05	Rheumatic mitral valve diseases

I06	Rheumatic aortic valve diseases

I07	Rheumatic tricuspid valve diseases

I08	Multiple valve diseases

**Target Group 12**	**Thyroid Disorders**

E00	Congenital iodine-deficiency syndrome

E01	Iodine-deficiency-related thyroid disorders and allied conditions

E02	Subclinical iodine-deficiency hypothyroidism

E03	Other hypothyroidism

E04	Other nontoxic goiter

E05	Thyrotoxicosis [hyperthyroidism]

E06	Thyroiditis

E07	Other disorders of thyroid

### Regression model

The unit of interest in the model is a country's vital registration system in a given year. Within each country-year, we are interested in the deaths related to heart failure, known as the heart failure universe deaths. The heart failure universe is defined as heart failure-attributed deaths (I50 or I50.0, I50.1, I50.9) and all possible targets included in the regression (Table 1), and is used as the denominator for the regression variables. The equation for the heart failure universe is as follows:

Each country-year will occupy a specific point along a spectrum, from all target group-attributed and no heart failure-attributed deaths (an ideal country-year) to no target group-attributed and all heart failure-attributed deaths (the worst-case scenario). Separate linear regressions were run for each target group to estimate the relationship across country-years between the proportion of heart failure-attributed deaths and the proportion assigned to each target group within the heart failure universe:

%TG = α + β[%HF] + ε, where

All analysis was run in STATA version 10.1SE. Separate regressions were run for each sex and for the following three age groups within each sex: 0-14, 15-49, 50+. Country-years were grouped into regions based on the GBD 2005 regions, and regions were stratified as developed or developing in accordance with the World Development Indicators 2008 [22]. Table 2 shows the 21 GBD regions stratified by development status. There were 338 developed country-years and 500 developing country-years used in the model. After the initial regression was run, target groups with statistically significant (p < 0.05) positive betas were dropped from the analysis. In these cases, the model made a statistical selection of the possible target groups previously chosen on the conceptual basis of heart failure pathophysiology. [Additional file 2 describes the interpretation of the regression results and the decision to drop specified targets.] By dropping a target, the heart failure universe decreases in size. The regression was rerun with the revised target group list and thus the smaller heart failure universe to ensure no changes in statistical significance in the revised heart failure universe. The process was repeated a second time. Ultimately, the constant values (y-intercept) yielded by the regression predict an ideal, all-target, no-garbage universe. The constant values for the target groups with statistically significant (p < 0.05) negative betas were scaled so they sum to 1 and are the redistribution proportions.

**Table 2:** Global Burden of Disease 2005 Regions, by development status

Developed	Developing
Asia Pacific, High Income	Asia, Central

Australasia	Asia, East

Europe, Central	Asia, South

Europe, Western	Asia, Southeast

North America, High Income	Caribbean

	Europe, Eastern

	Latin America, Andean

	Latin America, Central

	Latin America, Southern

	Latin America, Tropical

	North Africa / Middle East

	Oceania

	Sub-Saharan Africa, Central

	Sub-Saharan Africa, East

	Sub-Saharan Africa, Southern

	Sub-Saharan Africa, West

**A detailed list of GBD region country lists can be found in the GBD 2005 Operations Manual http://www.globalburden.org/gbdops.html

### Applying the redistribution proportions

Using the 2005 mortality data from the dataset, the redistribution proportions estimated by the above regression model were employed to redistribute all heart failure-attributed deaths to the appropriate target groups. Heart failure-attributed deaths for developed country adolescents (ages 0-14), for which no redistribution proportions were estimated, were redistributed to congenital heart anomalies. From the revised target groups, deaths were redistributed to the ICD-level causes comprising that target group, based on the relative prevalence of the ICD-level cause within that target group.

These corrected ICD-level causes were aggregated into GBD 2005 cause groups, and age-adjusted death rates by GBD cause were calculated on the pre- and post-redistribution datasets. Percentage change in age-adjusted death rate was calculated by cause. GBD-level causes of death were ranked by age-adjusted death rate in the pre- and post-heart failure redistribution datasets.

Table 3 outlines the seven major steps of the model and highlights assumptions at pertinent steps.

**Table 3:** Steps in redistribution of heart failure (HF)

	Details of step	Assumptions of step
Step 1	define pathophysiologically plausible target list at ICD level (these targets will ultimately receive redistributed HF deaths)	that only pathophysiologically plausible deaths are miscoded as HF

Step 2	group ICD-level causes into target groups of related causes^1^	

Step 3	choose representative mortality dataset (can be multinational or national depending on the population being examined)	that deaths coded to heart failure targets were correctly assigned

Step 4	use regression (%TG = α + β[%HF] + ε) to define redistribution proportions for each cause, by age-sex-development group^2^	that deaths miscoded as heart failure are miscoded at rates disproportionate to the relative prevalence of the underlying causes of heart failure

Step 5	redistribute deaths from HF to each target group by age-sex-development group within target mortality dataset	

Step 6	redistribute deaths from target group level to ICD cause level using proportionate redistribution^3 ^within target mortality dataset	

Step 7	use revised mortality dataset to calculate desired outcome measure [age-adjusted death rates, rank causes of death, etc.]	that there is no need to correct primary mortality dataset for completeness^4^

^1^table 1 shows the groups defined in this paper

^2^details of regression found in methods section and in additional file 2

^3^in proportionate redistribution, redistributions proportions are defined by the relative prevalence of the cause within the target group

^4^see discussion for an explanation of this assumption

## Results

Heart failure-attributed deaths comprise approximately 4.35 million deaths (3.1%) of the 142 million deaths in the dataset. Table 4 describes heart failure-attributed deaths by group in the dataset. Heart failure-attributed deaths are greater in older age groups and in developed country-years. Females from developed countries aged 50+ have the most heart failure-attributed deaths, with 36.9% of all heart failure-attributed deaths in the dataset. In the 15-49 age group, there are more heart failure-attributed deaths in developing country-years than in developed country-years - 4.7% and 1.1% of all heart failure-attributed deaths, respectively.

**Table 4:** Heart failure-attributed deaths by age, sex, development group, in thousands [percent of all heart failure deaths]

		Ages 0-14	Ages 15-49	Ages > 50
**DEVELOPED**	Males	1.2 [< 0.1]	34.6 [0.8]	981.5 [22.6]
	
	Females	1 [< 0.1]	13.7 [0.3]	1602.8 [36.9]

**DEVELOPING**	Males	24.6 [0.6]	129.1 [3.0]	704.8 [16.2]
	
	Females	20.8 [0.5]	74.5 [1.7]	758.1 [17.4]

**Figure 1:**
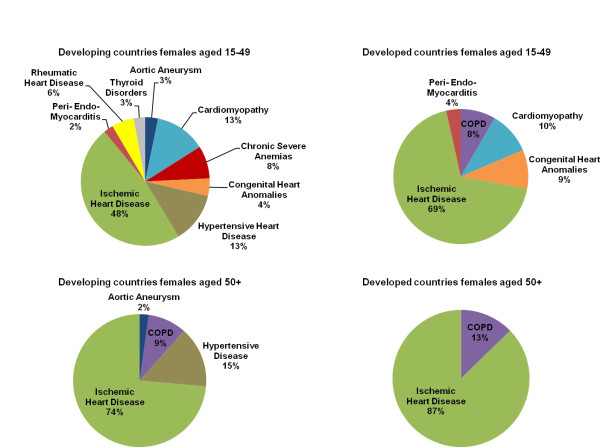
**Redistribution proportions by target group, females**. This series of pie charts displays the target groups, or underlying causes, for females by age and country development group that heart failure-attributed deaths are redistributed to and the proportion that each target group receives, as predicted by the regression model.

**Figure 2:**
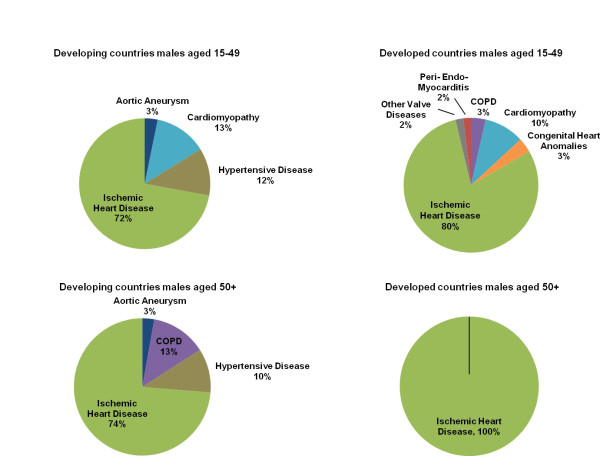
**Redistribution proportions by target group, males**. This series of pie charts displays the target groups, or underlying causes, for males by age and country development group that heart failure-attributed deaths are redistributed to and the proportion that each target group receives, as predicted by the regression model.

Figures 1 and 2 show a series of eight pie charts displaying the redistribution proportions estimated by the regression by sex and country development level for ages 15-49 and 50+. The proportions represent the fraction of heart failure-attributed deaths from that age-sex-country development group that should be redistributed to the specified target groups. Based on the statistical selection of targets in the model, certain target groups are not chosen for redistribution (β > 0, β ~0) for a given age-sex-country development group and are not found in the pie charts.

With 93.1% of all heart failure deaths in the dataset, the 50+ age group has the highest number of heart failure-attributed deaths. The model estimates that ischemic heart disease is the most important target group across both sex and country development stratifications in this age group. Collectively, ischemic heart disease has a higher redistribution proportion within the developed country-years compared to the developing country-years. [Additional file 3 displays the complete regression results for each target group for developing country males aged 50+.] Hypertensive heart disease is a redistribution target for both sexes in developing country-years (males = 0.104, females = 0.148) but is not a target in developed country-years.

With nine of the 12 targets being redistributed upon, the model predicts that developing country females of reproductive age (15-49) have the most diverse range of targets in comparison to all other age-sex-country development groups. This is the only group that has the following targets: chronic severe anemias, thyroid disorders, and rheumatic heart disease.

**Figure 3:**
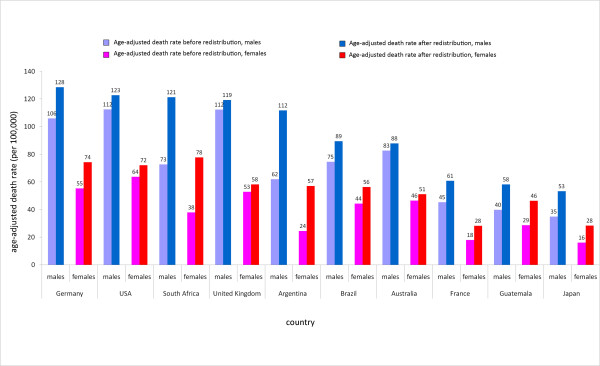
**Age-adjusted death rates (per 100,000) in 2005 for ischemic heart disease before and after heart failure redistribution, by country**. This graph shows the increase in age-adjusted death rates for ischemic heart disease for males and females in a series of 10 countries after redistribution of heart failure using the redistribution proportions predicted by the regression model, described in the preceding pie charts.

Redistributing heart failure using the above redistribution proportions on 2005 global mortality data provides the opportunity to assess the magnitude of change between a pre- and post-redistributed dataset. Figure 3 shows age-adjusted death rates for ischemic heart disease before and after heart failure redistribution for a selection of 10 countries. Age-adjusted death rates for ischemic heart disease are seen to be uniformly greater for males than females in both developed and developing countries. Argentine and South African males have the greatest absolute increase in age-adjusted death rates at 50 and 48 deaths per 100,000, respectively. This increase puts the age-adjusted death rate for South African males in the range of developed nations - two deaths greater per 100,000 than the United Kingdom and two deaths fewer per 100,000 than the US. With a greater than twofold increase, age-adjusted death rates for ischemic heart disease among South African females surpass several developed countries, including Germany, the US, and the United Kingdom.

**Figure 4:**
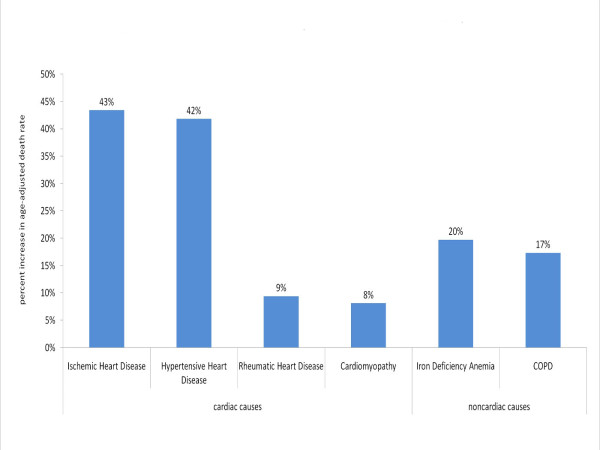
**Percent increase in age-adjusted death rate in 2005 in developing countries by cause, females**. This graph depicts the increase in age-adjusted death rates for several cardiac and noncardiac underlying causes of heart failure after the redistribution of heart failure-attributed deaths, using the redistribution proportions predicted by the regression model, for females in developing countries.

Figure 4 shows the percentage increase in age-adjusted death rates for pertinent causes in the group with the most diverse range of heart failure target groups - developing country women. This figure shows significant increases in heart failure causes associated strongly with pregnancy, including iron deficiency anemia and cardiomyopathy, a target inclusive of peripartum cardiomyopathy.

Table 5 shows a selection of countries in which there was a change in rank for the leading causes of death after heart failure redistribution. In France, ischemic heart disease surpasses cancer to become the top-ranked cause of death. In Japan, ischemic heart disease rises from the No. 3 to the No. 1 cause of death for men and No. 2 for women. Developing countries, such as Argentina and South Africa, see several important rank increases for hypertensive heart disease and COPD. COPD increases its rank in US females from No. 4 to No. 3, displacing Alzheimer's disease.

**Table 5:** Rank changes, by cause (bolded if cause breaks into top 15), 2005

		Ischemic heart disease rank	Hypertensive heart disease rank	COPD rank
Japan	Males	3 --> 1	46 --> 46	12 --> 12
	
	Females	2 --> 1	38 --> 38	32 --> 16

France	Males	2 --> 1	46 --> 46	12 --> 12
	
	Females	2 --> 1	28 --> 28	25 --> 16

Argentina	Males	1 --> 1	**24 --> 10**	7 --> 6
	
	Females	3 --> 1	**16 --> 6**	**18 --> 11**

South Africa	Males	5 --> 4	**16 --> 12**	7 --> 7
	
	Females	9 --> 5	11 --> 10	13 --> 12

***Ranks of GBD-level causes by age-adjusted death rate in the pre- and post-heart failure redistribution datasets

## Discussion

The use of heart failure as a cause of death code complicates the understanding of the major underlying causes of heart failure, resulting in a misallocation of limited health resources to prevent, detect, and treat those underlying conditions. Despite the WHO's recommendation to not use heart failure as an underlying cause of death, heart failure constitutes a considerable portion of global deaths and, as such, the redistribution of heart failure deaths to the appropriate underlying causes has a significant impact on causes of death at the population level. The benefit of the regression utilized in this model when compared to past models is that by comparing coding practices across country-years, it accounts for the fact that heart failure-attributed deaths are miscoded at rates *dis*proportionate to the relative prevalence of its underlying causes.

Consistent with the epidemiology of heart failure discussed in the literature [23-28], ischemic heart disease has the highest redistribution proportion for adults in all sex and country development stratifications. In developing countries such as South Africa, the significant increases in age-adjusted death rates highlight the epidemiologic transition, whereby disease burden shifts from communicable to noncommunicable diseases as countries become increasingly developed [29]. In developed countries where ischemic heart disease was not already the No. 1 cause of death, such as France and Japan, it became the top killer after heart failure redistribution. The relative increase in the age-adjusted death rate of ischemic heart disease in France - by 55% for women and 35% for men - prompts questions about the validity of the French paradox, an analysis using coronary heart disease as the cause of death variable without accounting for heart failure-attributed deaths [30]. Finally, even in countries such as the United Kingdom, with previously documented low miscoding rates [31], there is a notable increase in age-adjusted death rates.

The rise of hypertensive heart disease in developing country-years is likely due to a combination of the increasing burden of hypertension in developing nations coupled with the relative decreased access to antihypertensive medication in the developing world [32-34]. COPD is recognized in the literature as the most common cause of cor pulmonale [35], heart failure secondary to pulmonary disease, and represents the most significant noncardiac target for redistribution. The rank increases for hypertensive heart disease and COPD should bolster resources for hypertension control and pulmonary disease, with the latter an often-overlooked underlying cause of heart failure.

The complex epidemiological profile of reproductive-aged women (ages 15-49) in developing countries accounts for the most diverse range of heart failure targets compared to all other groups. In the absence of adequate antenatal care, including iron supplementation and routine monitoring, causes such as anemia, thyrotoxicosis, peripartum cardiomyopathy, and hypertension-induced heart disease become significant targets for heart failure redistribution [36-40]. Increased cardiac work in pregnancy will expose underlying cardiac conditions, such as congenital heart anomalies and rheumatic valve disease, which were less likely to have been diagnosed prior to pregnancy in developing countries [41]. This improved understanding of the underlying causes of heart failure deaths will enable clinicians and policymakers to better care for this vulnerable population.

There are several limitations of the model to consider. First, the *a priori *conceptual target list for the regression only includes targets that are pathophysiologically plausible causes of heart failure. There is no evidence to conclude that clinicians exclusively miscode heart failure deaths from pathophysiologically related underlying causes. The assumption regarding this issue is highlighted in Table 3, step 1. Additional file 1 lists the percent I50.9 (heart failure, unspecified) of all heart failure-attributed deaths, which may have a higher probability of being nonpathophysiologically related and thus would violate our assumption. Of note, there is a large variation in the percent I50.9 across countries [5.3-99.9%], and the average percent 150.9 in developed countries [54.6%] is roughly equivalent to that for developing countries [51.8%]. A recent paper by Stevens et al proposes a coarsened exact matching method to redistribute heart failure that includes nonpathophysiologically-related causes [42]. Future work could utilize this matching method to select targets for the regression method proposed in the paper. Second, as noted in Table 3, step 4, we assume that deaths coded to the defined underlying causes of heart failure have been correctly assigned. Third, there exists no gold standard with which to validate the results of the model. Field studies, such as the Puffer method, may offer some degree of validation [43]. However, even with a wide-scale autopsy study, it would be difficult to ascertain the etiology of heart failure-attributed deaths with certainty [16,44]. Finally, as highlighted in Table 3, step 7, we have assumed that the primary mortality datasets used in this model do not require correction for completeness. Previous literature has noted that countries have varying rates of data completeness and that correction for this incompleteness may enhance the comparability of national cause of death data [45]. When researchers or policymakers seek to replicate this model, they are encouraged to explore the completeness of their primary datasets. Despite these limitations, we believe that a rational interpretation of the results as found above is sufficient to confirm that the model provides the most viable method currently available to correct mortality data for heart failure.

This work highlights the need to improve the quality of death certification in all regions of the world. The generation of empirical evidence regarding the accuracy of death certification should be a future research priority. For example, field studies comparing registered causes of death with diagnoses on medical records or household-based verbal autopsies can shed some light on this issue. On a practical level, as electronic health records become increasingly utilized across the globe, installing safeguards to prevent the use of garbage codes in death certification is critical. Finally, educating health professionals to more accurately code underlying causes of death must be an essential component of any health system reform. This can be accomplished by not only providing better diagnostic tools and educating professionals about how to accurately certify deaths, but also by inspiring ownership and engagement in the crucial role of death certification in defining a health policy agenda.

## Conclusions

Despite an increase in both the quantity and quality of heart failure treatment modalities in recent years, mortality due to heart failure persists at a high rate [46]. This reality makes prevention efforts as well as early detection and treatment of the underlying conditions that lead to heart failure a top priority. The use of heart failure as an underlying cause of death obscures the true population causes of death, presenting a marked challenge to setting a health policy agenda that adequately rises to this challenge. The method described herein is systematic, replicable, and empirically based, allowing policymakers to maximize the use of available mortality data to determine these underlying causes of heart failure. Ultimately, this will enable policymakers to make more informed decisions regarding priority health interventions and resource allocation and thus hone the global response to the rising cardiovascular epidemic that has thus far been inadequate.

## Competing interests

The authors declare that they have no competing interests.

## Authors' contributions

RA, RL, MN, KF, EG, and CJLM designed the study. RA and KF performed statistical analyses. RA, RL, MN, KF, EG, and CJLM drafted the manuscript and approved the final version. CJLM accepts full responsibility for the work and the conduct of the study, had access to the data, and controlled the decision to publish.

## Funding Source

This research was supported by funding from the Bill & Melinda Gates Foundation.

http://www.gatesfoundation.org

The funders had no role in study design, data collection and analysis, decision to publish, or preparation of the manuscript.

## Supplementary Material

Additional File 1 **Countries in the dataset by GBD region, specifying the number and range of country-years as well as the development status**. This file is a table that lists the countries in the dataset by GBD region, specifying the number and range of country-years as well as the development status. The table also includes a column that describes the percent of heart failure-attributed deaths that were coded using ICD-10 code I50.9 (heart failure, unspecified) by country. Click here for file

Additional File 2 **Interpretation of regression employed in the model**. This file is a short explanation of how to interpret the regression method employed in the model. Click here for file

Additional File 3 **Complete regression results for developing country males, aged 50+**. This file displays the complete regression results for developing country males, aged 50+. Click here for file
