# Supplementary material for: Improving the public health utility of global cardiovascular mortality data: the rise of ischemic heart disease
Source: Popul Health Metr. 2011 Mar 15;9:8. doi: 10.1186/1478-7954-9-8 (PMC3064613; doi:10.1186/1478-7954-9-8)
Supplement: Additional File 1 — Countries in the dataset by GBD region, specifying the number and range of country-years as well as the development status. This file is a table that lists the countries in the dataset by GBD region, specifying the number and range of country-years as well as the development status. The table also includes a column that describes the percent of heart failure-attributed deaths that were coded using ICD-10 code I50.9 (heart failure, unspecified) by country. [file 1478-7954-9-8-S1.DOC]

**Additional File 1**

**Countries in the dataset by GBD region, specifying the number and range of country-years as well as the development status.**

**all data list the number of deaths due to each ICD code by country-year and stratified by age and sex

**percent I50.9 [I50.9 deaths / (I50.0 deaths + I50.1 deaths + I150.9 deaths)] applies only if data listed at the ICD-10 four-character level

| **COUNTRY** | **DEVELOPMENT STATUS** | **NO. OF YEARS [RANGE]** | **PERCENT I50.9** |
| --- | --- | --- | --- |
|  |  |  |  |
| **Asia Pacific (high income)** |  |  |  |
| Brunei Darussalam | Developed | 5 [1996-2000] |  |
| Japan | Developed | 13 [1995-2007] | 83.0% |
| South Korea | Developed | 12 [1995-2006] | 86.8% |
|  |  |  |  |
| **Australasia** |  |  |  |
| Australia | Developed | 9 [1998-2006] | 30.4% |
| New Zealand | Developed | 7 [2000-2006] | 15.3% |
|  |  |  |  |
| **Europe, Central** |  |  |  |
| Bulgaria | Developed | 1 [2006] |  |
| Croatia | Developed | 12 [1995-2006] | 93.9% |
| Czech Republic | Developed | 14 [1994-2007] | 81.0% |
| Hungary | Developed | 10 [1996-2005] | 58.6% |
| Poland | Developed | 8 [1999-2006] | 76.1% |
| Romania | Developed | 9 [1999-2007] | 74.8% |
| Serbia | Developed | 10 [1998-2007] |  |
| Serbia and Montenegro, Former | Developed | 6 [1997-2002] | 77.4% |
| Slovakia | Developed | 12 [1994-2005] |  |
| Slovenia | Developed | 11 [1997-2007] |  |
|  |  |  |  |
| **Europe, West** |  |  |  |
| Austria | Developed | 6 [2002-2007] | 94.6% |
| Belgium | Developed | 2 [1998-1999] |  |
| Cyprus | Developed | 5 [1998-1999, 2004-2006] | 84.1% |
| Denmark | Developed | 13 [1994-2006] | 77.1% |
| Finland | Developed | 12 [1996-2007] | 67.1% |
| France | Developed | 7 [2000-2006] | 64.2% |
| Germany | Developed | 9 [1998-2006] | 83.7% |
| Iceland | Developed | 12 [1996-2007] | 92.4% |
| Ireland | Developed | 1 [2007] | 25.5% |
| Israel | Developed | 9 [1998-2006] | 9.1% |
| Italy | Developed | 2 [2003, 2006] | 45.6% |
| Luxembourg | Developed | 9 [1998-2006] | 71.2% |
| Malta | Developed | 13[1995-2007] | 14.9% |
| Netherlands | Developed | 12 [1996-2007] | 73.6% |
| Norway | Developed | 11 [1996-2006] | 88.1% |
| Portugal | Developed | 4 [2002-2003, 2005-2006] | 65.0% |
| Spain | Developed | 7 [1999-2005] | 62.4% |
| Sweden | Developed | 10 [1997-2006] | 90.3% |
| Switzerland | Developed | 12 [1995-2006] |  |
| United Kingdom | Developed | 7 [2001-2007] | 15.5% |
| United Kingdom, England & Wales | Developed | 7 [2001-2007] | 15.0% |
| United Kingdom, Northern Ireland | Developed | 7 [2001-2007] | 13.6% |
| United Kingdom, Scotland | Developed | 8 [2000-2007] | 24.1% |
|  |  |  |  |
| **North America (high income)** |  |  |  |
| Canada | Developed | 5 [2000-2004] | 26.7% |
| Saint Pierre and Miquelon | Developed | 1 [2005] | 100% |
| United States of America | Developed | 8 [1999-2006] | 6.9% |
|  |  |  |  |
| **Asia, Central** |  |  |  |
| Armenia | Developing | 1 [2006] |  |
| Azerbaijan | Developing | 5 [2001-2004, 2007] |  |
| Georgia | Developing | 7 [1998-2001, 2004-2006] | 81.2% |
| Kazakhstan | Developing | 4 [2004-2007] | 53.5% |
| Kyrgyzstan | Developing | 7 [2000-2006] | 83.4% |
| Uzbekistan | Developing | 2 [2004-2005] |  |
|  |  |  |  |
| **Asia, East** |  |  |  |
| Hong Kong SAR | Developing | 7 [2001-2007] | 13.5% |
|  |  |  |  |
| **Asia, Southeast** |  |  |  |
| Malaysia | Developing | 6 [2000-2005] |  |
| Maldives | Developing | 6 [2000-2005] |  |
| Mauritius | Developing | 3 [2005-2007] | 15.3% |
| Rodrigues | Developing | 3 [2005-2007] | 5.3% |
| Réunion | Developing | 5 [2001-2005] | 60.1% |
| Seychelles | Developing | 5 [2001-2005] |  |
| Sri Lanka | Developing | 10 [1997-2006] |  |
| Thailand | Developing | 13 [1994-2000, 2002-2007] | 99.9% |
|  |  |  |  |
| **Caribbean** |  |  |  |
| Anguilla | Developing | 6 [2000-2001,2003-2006] | 46.2% |
| Antigua and Barbuda | Developing | 7 [2000-2006] | 18.3% |
| Aruba | Developing | 4 [1999, 2002-2004] | 94.6% |
| Bahamas | Developing | 4 [1999-2002] | 30.3% |
| Barbados | Developing | 4 [2000-2003] | 31.2% |
| Belize | Developing | 8 [1997-2004] | 57.1% |
| Bermuda | Developing | 7 [1996-2002] | 54.5% |
| British Virgin Islands | Developing | 8[1996-2003] | 37.5% |
| Cayman Islands | Developing | 6 [1998-2000, 2002-2004] | 36.8% |
| Cuba | Developing | 7 [2001-2007] | 28.1% |
| Dominica | Developing | 4 [2001-2004] | 36.6% |
| Dominican Republic | Developing | 8 [1996-2001, 2003-2004] | 46.6% |
| French Guiana | Developing | 5 [2001-2005] | 45.6% |
| Grenada | Developing | 5 [2001-2005] | 32.8% |
| Guadeloupe | Developing | 6 [2000-2005] | 58.8% |
| Guyana | Developing | 6 [2001-2006] | 23.1% |
| Haiti | Developing | 6 [1997, 1999, 2001-2004] | 67.6% |
| Martinique | Developing | 7 [2000-2006] | 53.9% |
| Montserrat | Developing | 9 [1995-2003] | 31.6% |
| Netherlands Antilles | Developing | 7 [1994-2000] | 71.3% |
| Puerto Rico | Developing | 6 [1999-2003, 2005] | 17.9% |
| Saint Kitts and Nevis | Developing | 10 [1996-2005] | 28.3% |
| Saint Lucia | Developing | 7 [1996-2002] | 55.2% |
| Saint Vincent and Grenadines | Developing | 5 [2000-2004] | 16.3% |
| Suriname | Developing | 11 [1995-2000, 2002-2006] | 81.9% |
| Trinidad and Tobago | Developing | 6 [1999-2004] | 44.5% |
| Turks and Caicos Islands | Developing | 10 [1996-2005] | 80.0% |
| Virgin Islands (USA) | Developing | 6 [1999-2003, 2005] | 11.9% |
|  |  |  |  |
| **Europe, East** |  |  |  |
| Belarus | Developing | 2 [2002-2003] |  |
| Estonia | Developing | 9 [1997-2005] |  |
| Latvia | Developing | 12 [1996-2007] |  |
| Lithuania | Developing | 10 [1998-2007] | 78.6% |
| Republic of Moldova | Developing | 12 [1996-2007] |  |
| Russian Federation | Developing | 8 [1999-2006] |  |
| Ukraine | Developing | 1 [2005] |  |
|  |  |  |  |
| **Latin American, Andean** |  |  |  |
| Ecuador | Developing | 11 [1997-2007] | 53.2% |
| Peru | Developing | 8 [1999-2006] | 67.1% |
|  |  |  |  |
| **Latin American, Central** |  |  |  |
| Colombia | Developing | 9 [1997-2002, 2004-2006] | 32.7% |
| Costa Rica | Developing | 11 [1997-2007] | 39.5% |
| El Salvador | Developing | 10 [1997-2006] | 12.7% |
| Guatemala | Developing | 2 [2005-2006] | 57.7% |
| Mexico | Developing | 10 [1998-2007] | 51.0% |
| Nicaragua | Developing | 9 [1997-2005] | 28.6% |
| Panama | Developing | 8 [1998-2004, 2006] | 45.0% |
| Venezuela | Developing | 10 [1996-2005] | 35.2% |
|  |  |  |  |
| **Latin America, South** |  |  |  |
| Argentina | Developing | 11 [1997-2007] | 87.5% |
| Chile | Developing | 11 [1997-2007] | 61.2% |
| Uruguay | Developing | 6 [1997-2001, 2004] | 69.0% |
|  |  |  |  |
| **Latin America, Tropical** |  |  |  |
| Brazil | Developing | 12 [1996-2007] | 37.8% |
| Paraguay | Developing | 9 [1996-2004] | 55.3% |
|  |  |  |  |
| **North Africa Middle East** |  |  |  |
| Algeria | Developing | 2 [2005-2006] |  |
| Bahrain | Developing | 5 [1997-2001] |  |
| Egypt | Developing | 5 [2003-2007] |  |
| Jordan | Developing | 3 [2004-2006] |  |
| Kuwait | Developing | 8 [1995-2002] | 99.9% |
| Oman | Developing | 3 [2005-2007] |  |
| Qatar | Developing | 1 [1995] |  |
|  |  |  |  |
| **Oceania** |  |  |  |
| Kiribati | Developing | 3 [1999-2001] | 85.7% |
|  |  |  |  |
| **Sub Saharan Africa, South** |  |  |  |
| South Africa | Developing | 10 [1996-2005] |  |
