# Supplementary material for: Improving the public health utility of global cardiovascular mortality data: the rise of ischemic heart disease
Source: Popul Health Metr. 2011 Mar 15;9:8. doi: 10.1186/1478-7954-9-8 (PMC3064613; doi:10.1186/1478-7954-9-8)
Supplement: Additional File 2 — Interpretation of regression employed in the model. This file is a short explanation of how to interpret the regression method employed in the model. [file 1478-7954-9-8-S2.DOC]

**Additional File 2**

**Interpretation of regression employed in the model**

The regression describes the relationship between heart failure-attributed deaths and target group-attributed deaths. The beta value signifies the rate of change of the proportion of the target group-attributed deaths within the heart failure universe as the proportion of heart failure-attributed deaths change across country-years. Three scenarios can occur with respect to the beta value:

- **Negative β**: The relative proportion of the target group-attributed deaths within the heart failure universe ***increases*** as the proportion of heart failure-attributed deaths decrease. Thus, deaths due to causes in this target group are being miscoded as heart failure deaths and should be redistributed to. The more negative the β value, the greater the frequency with which the target is attributed to heart failure.
- **β ~ 0**: The relative proportion of the target group-attributed deaths ***do not change*** as the proportion of heart failure-attributed deaths change. Thus, causes in this target group are not being miscoded to heart failure and should not be a target for redistribution.
- **Positive β**: The relative proportion of the target group-attributed deaths ***decreases*** as the proportion of heart failure-attributed deaths decrease. Thus, this target is not being miscoded to heart failure and should not be a target for redistribution.
