# Supplementary material for: Improving the public health utility of global cardiovascular mortality data: the rise of ischemic heart disease
Source: Popul Health Metr. 2011 Mar 15;9:8. doi: 10.1186/1478-7954-9-8 (PMC3064613; doi:10.1186/1478-7954-9-8)
Supplement: Additional File 3 — Complete regression results for developing country males, aged 50+. This file displays the complete regression results for developing country males, aged 50+. [file 1478-7954-9-8-S3.DOC]

**Additional File 3**

**Complete regression results for developing country males, aged 50+**

| **Target Group** | **Beta** | **Constant** | **p** | **N** | **Result** |
| --- | --- | --- | --- | --- | --- |
|  |  |  |  |  |  |
| **Regression 1** |  |  |  |  |  |
| Aortic_Aneurysm | -0.033 | 0.027 | 0.021 | 500 | Target |
| COPD | -0.092 | 0.124 | 0.000 | 500 | Target |
| Cardiomyopathy | -0.016 | 0.031 | 0.072 | 500 | Non-significant |
| Chronic Severe Anemias | -0.003 | 0.003 | 0.371 | 500 | Non-significant |
| Congenital Heart Anomalies | 0.000 | 0.001 | 0.424 | 500 | Non-significant |
| Hypertensive Heart Disease | -0.068 | 0.098 | 0.006 | 500 | Target |
| Ischemic Heart Disease | -0.764 | 0.696 | 0.000 | 500 | Target |
| Other Respiratory Diseases | 0.001 | 0.001 | 0.476 | 500 | Non-significant |
| Other Valve Diseases | -0.006 | 0.007 | 0.180 | 500 | Non-significant |
| Perio- Endo- Myocarditis | 0.004 | 0.001 | 0.000 | 500 | Drop |
| Rheumatic Heart Disease | -0.002 | 0.004 | 0.140 | 500 | Non-significant |
| Thyroid Disorders | -0.020 | 0.008 | 0.130 | 500 | Non-significant |
|  |  |  |  |  |  |
| **Regression 2** |  |  |  |  |  |
| Aortic_Aneurysm | -0.033 | 0.027 | 0.021 | 500 | Target |
| COPD | -0.092 | 0.124 | 0.000 | 500 | Target |
| Cardiomyopathy | -0.016 | 0.031 | 0.075 | 500 | Non-significant |
| Chronic Severe Anemias | -0.003 | 0.003 | 0.373 | 500 | Non-significant |
| Congenital Heart Anomalies | 0.000 | 0.001 | 0.430 | 500 | Non-significant |
| Hypertensive Heart Disease | -0.068 | 0.098 | 0.005 | 500 | Target |
| Ischemic Heart Disease | -0.761 | 0.696 | 0.000 | 500 | Target |
| Other Respiratory Diseases | 0.001 | 0.001 | 0.473 | 500 | Non-significant |
| Other Valve Diseases | -0.006 | 0.007 | 0.180 | 500 | Non-significant |
| Rheumatic Heart Disease | -0.002 | 0.004 | 0.142 | 500 | Non-significant |
| Thyroid Disorders | -0.019 | 0.008 | 0.131 | 500 | Non-significant |
|  |  |  |  |  |  |
| **Regression 3** |  |  |  |  |  |
| Aortic Aneurysm | -0.033 | 0.027 | 0.021 | 500 | Target |
| COPD | -0.092 | 0.124 | 0.000 | 500 | Target |
| Cardiomyopathy | -0.016 | 0.031 | 0.075 | 500 | Non-significant |
| Chronic Severe Anemias | -0.003 | 0.003 | 0.373 | 500 | Non-significant |
| Congenital Heart Anomalies | 0.000 | 0.001 | 0.430 | 500 | Non-significant |
| Hypertensive Heart Disease | -0.068 | 0.098 | 0.005 | 500 | Target |
| Ischemic Heart Disease | -0.761 | 0.696 | 0.000 | 500 | Target |
| Other Respiratory Diseases | 0.001 | 0.001 | 0.473 | 500 | Non-significant |
| Other Valve Diseases | -0.006 | 0.007 | 0.180 | 500 | Non-significant |
| Rheumatic Heart Disease | -0.002 | 0.004 | 0.142 | 500 | Non-significant |
| Thyroid Disorders | -0.019 | 0.008 | 0.131 | 500 | Non-significant |
